# Security Requirements for the Internet of Things: A Systematic Approach

**DOI:** 10.3390/s20205897

**Published:** 2020-10-19

**Authors:** Shantanu Pal, Michael Hitchens, Tahiry Rabehaja, Subhas Mukhopadhyay

**Affiliations:** 1 Department of Computing, Faculty of Science and Engineering, Macquarie University, Sydney, NSW 2109, Australia; shantanu.pal@hdr.mq.edu.au (S.P.); michael.hitchens@mq.edu.au (M.H.); 2 Macquarie University Cyber Security Hub, Faculty of Science and Engineering, Macquarie University, Sydney, NSW 2109, Australia; tahiry.rabehaja@mq.edu.au; 3 School of Engineering, Faculty of Science and Engineering, Macquarie University, Sydney, NSW 2109, Australia

**Keywords:** Internet of Things, access control, threats and attacks, security requirements

## Abstract

There has been a tremendous growth in the number of smart devices and their applications (e.g., smart sensors, wearable devices, smart phones, smart cars, etc.) in use in our everyday lives. This is accompanied by a new form of interconnection between the physical and digital worlds, commonly known as the Internet of Things (IoT). This is a paradigm shift, where anything and everything can be interconnected via a communication medium. In such systems, security is a prime concern and protecting the resources (e.g., applications and services) from unauthorized access needs appropriately designed security and privacy solutions. Building secure systems for the IoT can only be achieved through a thorough understanding of the particular needs of such systems. The state of the art is lacking a systematic analysis of the security requirements for the IoT. Motivated by this, in this paper, we present a systematic approach to understand the security requirements for the IoT, which will help designing secure IoT systems for the future. In developing these requirements, we provide different scenarios and outline potential threats and attacks within the IoT. Based on the characteristics of the IoT, we group the possible threats and attacks into five areas, namely *communications*, *device/services*, *users*, *mobility* and *integration of resources*. We then examine the existing security requirements for IoT presented in the literature and detail our approach for security requirements for the IoT. We argue that by adhering to the proposed requirements, an IoT system can be designed securely by achieving much of the promised benefits of scalability, usability, connectivity, and flexibility in a practical and comprehensive manner.

## 1. Introduction

There has been tremendous growth in the use of the Internet of Things (IoT) [1] in our daily lives. The IoT enhances Web-enabled applications, where ‘everyone’ (e.g., people) and ‘everything’ (e.g., systems, machines, equipment, devices, etc.) in a physical or virtual world can be connected over the Internet [2]. The rapid growth in the number of smart devices (e.g., smart phones, tablets, intelligent circuits, sensors, actuators, etc.) makes it more convenient to use IoT applications and services than ever before [3]. It is predicted that the number of devices connected to Internet will be 28.5 billion in 2022, up from 18 billion in 2017 [4]. On an individual basis this will mean 3.6 networked connected devices per capita by 2022, compared to 2.3 in 2016. The average number of devices and connections per household and per Internet user is predicted to increase by 51% by 2022. This trend will also increase the annual global Internet traffic, which is predicted to reach 4.8 ZB (zetta-bit) per year by 2022 [4]. This signifies the potential scale of the IoT where billions of *things* will be connected via the network. With an increase in scale comes an increase in the value of the data stored, processed and transferred and the attacks upon them. In other words, these forecasts indicate that the number and sophistication of attacks and threats against these embedded devices will continue to rise and therefore greater security measures are needed [5,6,7]. In such, how to protect the sensitive information from unauthorized users and services by considering the core security requirements when designing an IoT architecture is a significant issue. Note, for our purposes, a *thing* is one or a set of users, devices, services and applications, and similar entities.

### 1.1. Problem Statement

The term ‘IoT’ was popularized by the innovative work of the Massachusetts Institute of Technology (MIT) Auto-ID Centre. The first documented evidence of the use of the term ‘The Internet of Things’ was by Kevin Ashton, the co-founder of the MIT Auto-ID Centre, in the year 1999 [8]. The IoT connects all the devices in a physical domain with the Internet to communicate with each other for faster and easier service. The IoT represents a view in which the traditional Internet extends into real-world objects (e.g., food, clothing, furniture, paper, landmarks, refrigerator, etc.) and enables each object with the ability to gather, process and act on information in a *smarter* way. These objects, acting as sensors or actuators, are able to interact with each other in order to reach a common goal (e.g., quality and service) by connecting all smart *things* to the current Internet [9]. Therefore, the prospect for the IoT is to deploy a ubiquitous environment where the subjects (i.e., people) and the objects (i.e., resources), that are addressable and communicable, will be connected over a network platform to leverage the benefits for both society and technology on a large-scale, so that human users are unobtrusively assisted by technology in performing everyday activities [10].

Major issues for a wider deployment of IoT systems include: limited storage and processing capacity of the *things*, concerns regarding reliability in performance, availability in communication mediums, accessibility any-time and any-where, interoperability in a heterogeneous environment, data management performance and security and privacy [11,12,13,14,15,16]. Due to the resource-constrained nature of the IoT devices, it is hard to enforce traditional heavy-weight security mechanisms within these devices. Moreover, due to the inadequate physical security of the *things*, they can be easily attacked [17]. For example, unauthorized access to the resources (both service and network) can be carried out over unsecured wireless connections [18]. Examples of such attacks include targeting the control of IoT-enabled automobile systems (for example, remotely attacking an automatic car) [19] and hacking critical IoT-enabled medical devices (for example, altering the dosage of drugs administered to a patient after remotely controlling the drug infusion pumps) [20]. In 2016, an attack called ‘Mirai Botnet’ [21] infected numerous IoT devices (in particular older routers and IP cameras) then flooded dynamic DNS service provider with network traffic via a DDoS (Distributed Denial-of-Service) attack. This particular attack illustrates that IoT devices have been lagging behind in terms of security. In fact, the hacker’s main entrypoints into these devices were by using default hardcoded factory username and passwords. In Finland, in 2016, attackers gained unauthorized access to the systems controlling facilities in an apartment and shut down the central heating and hot-water systems [22].

This summary indicates the need to protect IoT systems and resources from potential threats and attacks not only in internal networks but also originating from networks that span multiple jurisdictions. With the sensitive nature of the IoT and its dynamic characteristics, many of these issues cannot be addressed with a simple software patch or commonly used security measures [23,24,25]. Security solutions for the IoT need to be designed for their intended context, the IoT. Enforcing security policies and developing appropriate security requirements for the IoT has not only become an essential issue but also an obligation. In this paper, our intention is to investigate the security requirements that need to be met by any proposed solution for a secure IoT system. Note, a comprehensive security analysis for an IoT system (e.g., vulnerabilities, attacks mechanisms and their countermeasures) is omitted in this paper, we direct interested readers to the specific security analysis papers cited in various sections.

### 1.2. Contributions

The IoT enables the connection of a very large-scale quantity of devices, users and their associated services and applications, enabling them to interact with one another. While this improves the users’ experience, it also poses multiple security issues [26,27,28]. Numerous proposals to address the security issues of the IoT have been advanced and address areas such as access control, privacy, trust, identity, etc. [29,30,31]. Most of these focus on solutions to individual issues. A few surveys, e.g., [32,33,34], address the security issues of an IoT system in terms of a layer-based approach (i.e., security specific requirements in each architectural layer) and discuss the requirements of each layer individually. These approaches explore the security requirements against commonly known metrics e.g., resource limitation, fault tolerance, heterogeneity, authentication, confidentiality, access control and do not provide a detailed requirement for a large-scale dynamic system such as the IoT.

Several works discuss IoT applications and different frameworks based on technology, issues and applications [35,36,37,38,39]. Others list the system challenges when integrating cloud and IoT-based applications [40]. While a few of them (e.g., [41]) briefly outline some security requirements, most of them lack a justification for the security requirements presented, particularly in the context of the integrated services, applications and domains of the IoT.

In [42] the authors discussed various security challenges for the IoT and state of the art efforts to resolve these challenges. The security challenges discussed include privacy, light-weight cryptographic framework, secure routing, robustness and resilience management, and insider attack detection. However, how these challenges help to constitute appropriate security requirements for the IoT systems is not discussed in this proposal. With a similar view to [42,43] presented a comprehensive study listing security issues and corresponding defence mechanisms for the IoT. Through an empirical study, it attempted to deliver an insight into the security requirements of IoT systems. However, the contributions are limited to the IoT security issues and without a detailed discussion on the actual security requirements needed for an IoT system.

To the best of our knowledge, as of today, there is no complete set of requirements for an IoT security architecture that fully addresses all the needs of an IoT system [30,44]. This creates significant challenges for the secure, robust, and scalable deployment of IoT applications and services. We note, the present security architectures do not adequately identify and integrate the characteristics and security-specific requirements of an IoT system. The wider scale of the IoT systems and the vast range of applications will require a security architecture whose design must take full account of different characteristics of such sysytems [45]. In this paper, we try to bridge the gap by synthesizing the existing proposals of the state of the art security requirements for the IoT in a single manuscript. In particular, we aim to address the following key research question: *what are the requirements for the design and provision of security for the IoT?*

Most of the aforementioned proposals do not differentiate between various security challenges and security requirements for the IoT. In other words, they do not discuss the technical issues and characteristics required to derive the list of security requirements for an IoT system. While a few of them address the requirements on an individual level, they do not focus on a comprehensive survey. Some surveys also ignore important IoT issues e.g., self-healing and openness. We intend to examine the critical security requirements for the IoT mainly by surveying the existing state of the art in this field, and compare and contrast the available proposals with one another. In this paper, our approach is three-fold: (1) we outline the potential threats and attacks in an IoT system, (2) we examine the available security requirements for the IoT, and (3) we study and analyse how those requirements can be employed to design a secure IoT architecture. While the list of works discussed above provides a basic foundation for understanding security requirements of the IoT, our survey differs from previous proposals in many ways. To the best of our knowledge, this survey provides the most comprehensive and the detailed discussion on the security requirements of an IoT system. In particular, the major contributions of this paper are:

- We examine and map the various threats and attacks to an IoT system into five distinct areas, namely communications, device/services, users, mobility and integration of resources. This helps to guide the derivation of unique security requirements for the IoT.
- Compared to the existing proposals in the field of the IoT security, we provide a comprehensive discussion of the IoT security requirements in a systematic way, and present a critical discussion of the employments of such requirements to design an IoT security architecture.
- Based on the investigation, we indicate the employment of specific security requirements for the each layer of an IoT security architecture. Our approach considers both the technological and architectural point of views of an IoT system.

### 1.3. Organization and Roadmap

The rest of the paper is organized as follows. In Section 2, we present a brief introduction of IoT. This covers various definition of IoT. In Section 3, we discuss a generic IoT security architecture and provide an outline of different architectural layers. We briefly discuss some example application areas for the IoT in Section 4. In Section 5, we list various threats and attacks exist in an IoT system in a systematic way. This strategically covers the various potential threats and attacks in an IoT system into five distinct areas. We present related works in the state of the art IoT security requirements in Section 6. We present our approach to IoT security requirements in Section 7. In Section 8, we summary the lessons learned and discuss the various security requirements for different layers in an IoT architecture. Finally, we conclude the paper in Section 9.

## 2. The IoT Paradigm

The IoT is not a single system composed of computing devices. It is more of a system interconnected to various smart objects (both physical and digital) that encompasses everything and anything connected to the Internet [46]. There are several definitions of the IoT that have been presented. For instance, according to the Information Society and Media Directorate-General of the European Commission (DG INFSO) and the European Technology Platform on Smart Systems Integration (EPoSS), IoT is defined as [47]: *“things having identities and virtual personalities operating in smart spaces using intelligent interfaces to connect and communicate within social, environmental, and user contexts”*. This is a widely used IoT definition that follows a *things* oriented architecture. Furthermore, Atzori et al. [1] define *things* from three perspectives e.g., middleware service, sensors and information.

Buyya et al. [48] present a user-oriented definition of the IoT regardless of communication protocols and IoT environments: *“interconnection of sensing and actuating devices providing the ability to share information across platforms through a unified framework, developing a common operating picture for enabling innovative applications. This is achieved by seamless ubiquitous sensing, data analytics and information representation with cloud computing as the unifying framework”*.

Compared to [1,48], Tan and Wang [49] define the IoT from the viewpoint of communication, social, environment and user contexts, as follows: *“things have identities and virtual personalities operating in smart spaces using intelligent interfaces to connect and communicate within social, environment, and user contexts”*.

Unlike the approaches by [1,48,49], Haller et al. [50] defined the IoT independently of technology and platforms. This definition is derived from a mobility and service integration perspective. In addition to other researchers, Davoli et al. [51] discussed the IoT from a network point of view, where the physical devices are connected in an Internet-like structure. A list of other definitions for the IoT can be found in [52].

In summary, the IoT is not just a cyber-physical system for measuring state information and doing automatic computation. It is more of a networking infrastructure that combines the digital and physical worlds together. Therefore, we argue that when we address the security characteristics of IoT, we need to consider a wider aspect of scenarios combining architectures, users, communications, technologies and applications. Furthermore, in the IoT, the interactions between the entities may occur for a short period of time, and maybe only once in their lifetime. It is also challenging to predict, in advance, which entities will interact to which entity and to specifically identify the definite services to which they will attempt access. In an IoT system, where users may have a large number of devices and their associated services, being able to efficiently identify them, both uniquely and as groups, is crucial to ensure the security and privacy of the system. Therefore, we further argue that in a highly scalable and dynamic system such as the IoT, the authentication of an entity must not be dependent upon the unique concrete identity of the entity. This could be best represented as a set of ‘attributes’ that can help to reduce the overhead on the system by avoiding the need to store and specify policies based on the identity of each entity. Attributes can be the name, age, location or an activity that describe an aspect of an entity in a specific context [53].

## 3. IoT Security Architecture

The goal of this section is to discuss an IoT security architecture. There are several IoT architectures proposed in the literature, for example [54,55,56,57,58]. Many of them proposed a three-layer architecture (e.g., [59,60,61]) composed of application, network and perception layers. A few of them (e.g., [44]) propose a four-layer architecture consists of sensing layer, network layer, service layer and application-interface layer. However, there is no generic architecture for the IoT that has converged to a commonly used reference model. In contrast to the three and four layers architectures, Ref. [56] argues for the support for a five-layer architecture for IoT applications and services. The layers used there are, from bottom to top, objects, object abstractions, service management, application and business. While we agree that multiple layers are necessary to capture the complexity of the IoT, we consider that this particular model glosses over the details of the physical structure of such systems.

Previous work on security (e.g., [44]) has followed the layered architecture approach for the IoT. However, the approach taken has typically been fairly simplistic, dividing security functionality between the layers. This ignores both the complexity of the IoT and the need to provide similar security functionality in different places and in different forms. For example, authentication will be needed both for individual devices and for applications. However, whether the same mechanism, or even the same credentials, could be used in both cases is unclear in a general sense. Note, this is dependent on the specific architecture of each system, and therefore, it is not explicitly stated. Therefore, we propose a model which is layered in both the horizontal and vertical planes. The horizontal planes cover the architectural components of the IoT, from devices, through the connecting network to service composition up through applications to the end-user. The vertical planes cover security services, e.g., authentication, authorization, identity management, trust management, key management, etc. As noted above, these may be required at various system architecture levels. Even if the mechanisms at each level are not the same, they will need to inter-operate.

**Figure 1:**
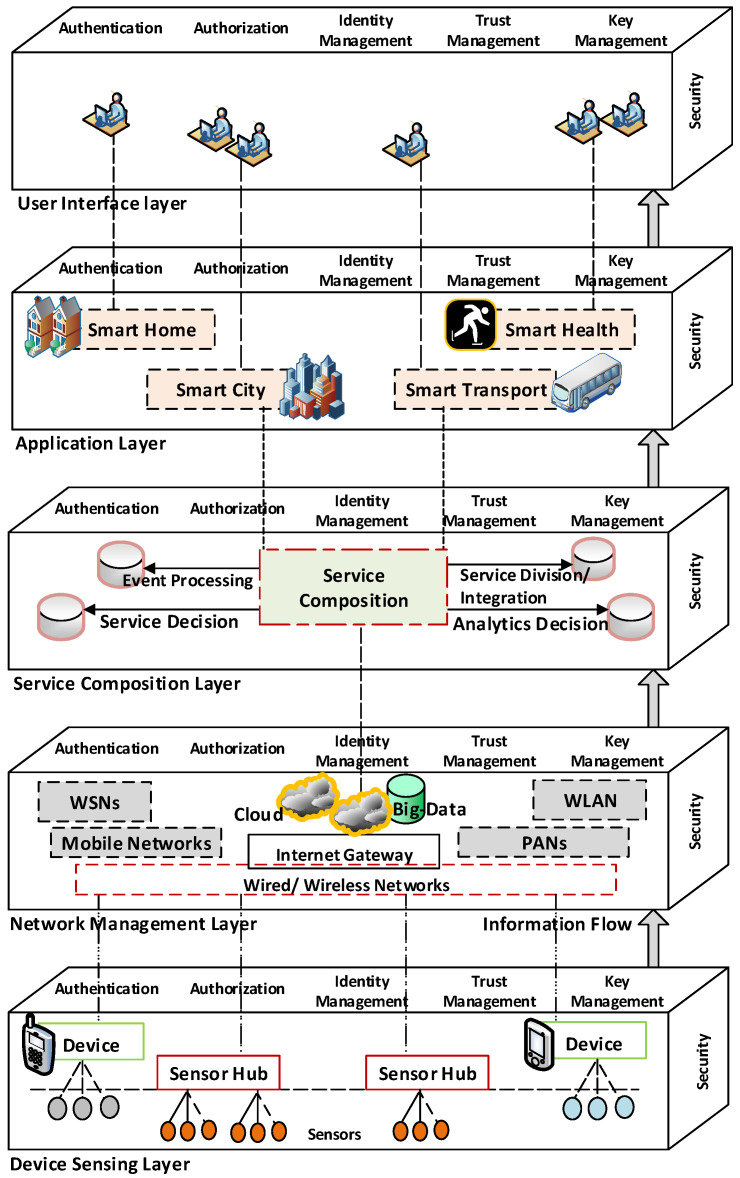
The functional layers of an IoT security architecture.

We argue that the functional components of an IoT architecture should encapsulate the diverse security requirements and various security issues of this context [62]. The architecture should enable the achievement of security for devices, networks, data repository, services, applications and users. Therefore, based on analysis of the previous works, we suggest a five-layer, three-dimensional IoT security architecture (cf. Figure 1). However, unlike the other architectures (e.g., [44]), where users and applications reside in the same layer, we separate the users from the application layer and situate them on the top of it. This will better help to scale the vast amount of users in a large-scale IoT system and address user-specific security issues. Note, our architecture is superficially similar to [44] (which has four layers, namely sensing, network, service and interface) and supports the arguments discussed in [56]. To the layers of the architecture, we add another dimension to explicitly include the need for core security functionality at each layer of the architecture. Essentially the architecture employed here is that of [44], with the final layer divided into users and application to better represent their individual needs and security issues and, more importantly, the addition of the extra dimension of security.

The five layers (bottom-up) are: *device sensing layer*, *network management layer*, *service composition layer*, *application layer* and *user interface layer*. Each layer contains the architectural elements that are necessary to collect, store, compute, process and communicate information between the architectural elements and between the layers. To the plane these layers constitute we add another, consisting of some basic security requirements e.g., *authentication*, *authorization*, *identity management*, *trust management* and *key management*. Please note that this list of basic security requirements is not exclusive and will likely need extending. The results is a horizontal plane delivering the security required at each level and a vertical plane consisting of system functionality. We now present a brief outline of each layer in the vertical plane. This includes the core components, major functionalities and common security issues for each layer.

### 3.1. Device Sensing Layer

The first layer is composed of smart IoT sensing devices e.g., smart phones, RFID tags, sensors and actuators, etc. These components are able to automatically sense, collect and measure the various physical parameters e.g., temperature, humidity, location etc. Devices can store collected information inside themselves and sensors can store information into predefined sensor hubs (e.g., a microcontroller unit) to process them.

The major functionalities of this layer are data sensing and data acquisition. Standardized plug-and-play mechanisms can be used with the various sensing devices. Furthermore, considering the scale of the number of *things* in an IoT system, sensing devices may be deployed simultaneously or over time according to the environmental context and practical requirements [63]. Regardless, security is an important issue in their deployment and use. Common security issues in this layer include authentication of the *things* (i.e., sensing devices in general), authorization and access control as well as the availability of infrastructure and networks for a seamless integration of *things* for data access.

### 3.2. Network Management Layer

The second layer is the network management layer. This layer is composed of different wired and wireless networks, cloud computing services and big data repositories. Major functionalities of this layer include data aggregation, Quality of Service (QoS), scheduling, etc. It is also responsible for transmitting data to the next IoT architectural layer. The networks in this layer potentially combine heterogenous equipment and help to transmit data among different components within this layer (and to the next architectural layer) using technology including 3G, 4G, GSM (Global System for Mobile Communication), UMTS (Universal Mobile Telecommunications System), WiFi, Bluetooth, etc. The presence of cloud computing services and big data repositories enable a variety of different technologies to perform seamlessly by deploying, managing and scheduling of various network services [64]. Other commonly used technologies in this layer are IPv6, 6LoWPAN (IPv6 over Low-Power Wireless Personal Area Networks), and RPL (IPv6 Routing Protocol for Low-Power and Lossy Networks). With the recent advancement, 6LoWPAN is a dedicated communication protocol in this layer that can fit well with the resource-constrained IoT devices. 6LowPAN is designed for IPv6 over IEEE 802.15.4. Similarly to 6LoWPAN, RPL facilitates communication in a resource-contained environment and specifically within constrained networks, e.g., wireless sensor networks [65,66]. Some common security issues in this layer include unauthorized access to sensitive information, modification of routing paths or even an attempt to make the IoT resource unavailable to the users by congestion of communication channels by Denial of Service (DoS) attacks.

### 3.3. Service Composition Layer

The third layer is the service composition layer. The major functions of this layer are analysis and processing of data that is collected from the network management layer. The service composition layer is built based on middle-ware technology that assists with information exchange for IoT applications among heterogeneous objects without any specific hardware and software requirements. It is intended to meet the needs of applications, application programming interfaces (APIs) and various service protocols [67]. The major functional component of this layer is the service composition unit, which is responsible for event processing, creating service divisions, service monitoring, service configuration and performing various decision analytics according to the specific policy requirements and contextual information. Common security issues in this layer include service (or group) authentication, data confidentiality (includes leakage of private information from various data sources) and integrity.

### 3.4. Application Layer

The fourth layer is the application layer which provides smart IoT services to users. The major functional components of this layer are various applications which could be classified as, for example, smart home, smart city, smart transport, smart commerce and smart health, etc. [67,68,69] (cf. Section 4). The application layer is responsible for providing various services and at the same time determines a set of massage passing protocols at the application level [70]. This layer is also responsible for data presentation, application maintenance, application access control and updating software and security patches for those applications. Standard interfaces using HTTP and HTTPS are widely deployed for this layer. However, more dedicated resource constrained application level protocols e.g., CoAP (Constrained Application Protocol), Message Queue Telemetry Transport (MQTT), Advanced Message Queuing Protocol (AMQP), eXtensible Messaging and Presence Protocol (XMPP), etc. are also used in this layer [71,72]. Common security issues in this layer include unauthorized use of access services, privacy leakage, resource unavailability, etc.

### 3.5. User Interface Layer

The fifth and final layer is the user interface layer. The interface provided to users and the *users* themselves are the major functional components in this layer. This layer exports the system’s functionalities from the application layer to the end-users. It may use standard Web services (both for service protocol and service composition) to distribute the activities and services received from the application layer [32]. Common security issues in this layer include authentication and authorization of users, unauthorized data access, data confidentiality, availability of services, etc.

Please note that the different layers have distinct security requirements based on the core security functionalities. For instance, key management in the device sensing layer deals with confidentiality, whereas it may also deal with integrity in the service composition layer. Similarly, identity management in the device sensing layer protects service privacy; however, it safeguards users’ privacy in the user interface layer. In Table 1, we illustrate the core components, major functionalities and common security issues for each layer discussed above.

## 4. IoT Applications

The motivation of this section is to discuss various application areas of IoT. Numerous applications and services can be and have been employed in the IoT [33,73,74]. Here we outline a few of them, detailed descriptions of them can be found in the cited works. Note, before discussing IoT security threats and attacks and the security requirements in detail, in this section, we aim to present these example application areas for the IoT within which various threats and attacks may occur.

### 4.1. Smart Healthcare

With the rapidly increasing deployment of WSNs, RFID, smart wearable devices and sensors (e.g., Fitbit [75]), healthcare systems are relying more and more heavily on IoT-enabled smart applications [35,76,77,78,79,80]. In such smart healthcare systems, patient monitoring and administration of appropriate medication can be controlled and managed automatically without any direct human involvement. In the past, healthcare systems were a closed environment within a secure network infrastructure. However, with the IoT they are now operating in an open context [81]. For example, using wearable blood pressure monitoring systems, a patient’s data (i.e., blood pressure) can be periodically transferred to the hospital database and viewed by appropriate doctors. It could then be used for diagnosis and treatment-plan purposes. For instance, using ‘BioStrap’ [82], a wearable wrist-band and shoe clip to monitor heart rate, a user’s medical data (e.g., heart rate, blood oxygen saturation level or sleeping analysis) can be monitored and stored appropriately. This device can be controlled and monitored using smart phone applications.

### 4.2. Smart Home and Buildings

Smart home is intended to provide a more flexible and comfortable life-style with IoT-enabled home appliances [69,83]. For example, intelligent sensors can attempt to gauge a person’s emotional state from physiological readings and change the environment of a room accordingly. A smart electronic heater can adjust the temperature of a room automatically without any human intervention. A smart electric meter can automatically send readings to the billing company. There are many actual applications available in the market, for example, the ‘CURB’ [84] energy intelligence system, which allows users to automatically adjust the temperature of a home remotely. It can also detect which devices are turned on in a particular time-frame and how much power they are using. Based on such data, it can predict future utility costs. Another example is the ‘Philips Hue’ [85] wireless lighting system, where a user can control the lights using their voice, adjust the brightness, set timers, create routines or even can change colours using a mobile app.

### 4.3. Smart Transportation

This is also referred to as the intelligent transportation system. In addition to controlling or supporting the vehicles themselves, it helps to monitor and control traffic data (between the vehicles and the transportation infrastructure), compute and integrate this data in real-time, as well as communicate with the transportation networks for analysis and evaluation purposes. It typically involves GPS and RFID based tracking systems [86,87,88]. For instance, ‘B-Scada’ [89], an IoT-enabled system-wide data management infrastructure used for smart transportation systems, collects real-time data from different sources, performs analysis and implements appropriate solutions, e.g., redirect traffic routes, etc. With the IoT, scheduling and cargo distribution and fuel consumption can also be improved in terms of efficiency and cost [90].

### 4.4. Smart Grid

Smart grid is an example of smart infrastructure that supports electricity distribution, management and consumption. It includes a variety of operational and energy measures including smart meters, smart appliances and various energy efficient applications [91]. Smart grid systems encompass intelligent distribution and control systems from the central core to the edge networks. This will help meet the demand for improved energy efficiency via low cost and low powered IoT devices. Several projects (e.g., [92]) are also aimed at reducing carbon emissions and achieving high energy efficiency [93,94,95].

### 4.5. Smart City

A smart city can be viewed as the ubiquitous systems of various IoT-enabled applications and services (e.g., health, buildings, transportation, utilities, etc.) that are combined to serve a large urban area [96,97,98,99]. The vision is to create an environment (incorporating information and communication technologies) that will improve the quality of city-life for people living and working in the city and provide improved interactions between various entities, systems and applications [86]. At the same time, it will help manage the economy, environment, mobility and governance of city infrastructure and services [100]. There are several initiatives that have been taken to provide IoT-enabled smart cities. For instance, ‘Smart Nation Singapore’ [101], ‘Amsterdam Smart City’ [102] and ‘Barcelona Smart City’ [103]. These initiatives provide real-life smart city experiences through sustainable spatial development, smart digital connectivity and enriched connected IoT services.

## 5. Threats and Attacks

In this section, we examine the potential threats and attacks for the IoT, including the various application scenarios that we discussed above (in Section 4). There have been several works that discussed IoT security and examined threats and attacks therein [34,37,104,105,106,107,108]. Many works, e.g., [32,44,109,110,111,112,113,114], categorize potential threats and attacks based on the different layers of an IoT architecture. Some of them (e.g., [30,115]) derive threats and attacks based on particular security issues e.g., identity, access control, trust, middleware and mobility. A few of them (e.g., [116,117]) also categorize threats and attacks based on the applications and specific use-case scenarios. Furthermore, Ref. [45] categorizes various security issues in an IoT system based on the nature of the IoT infrastructure e.g., centralized, collaborative, connected and distributed IoTs. However, we argue that the classification is not clear and nor do they address the differences between the various attack scenarios that exist in the IoT and traditional distributed systems. The IoT presents its own distinct challenges in composing secure and trustworthy solutions. The scale and nature of the IoT systems make it difficult to specify a threat and attack model by using commonly used security mechanisms. Therefore, it is hard to employ established threat modeling methods, for instance, used for general computing systems, in the IoT. Next, we address various aspects of the IoT environment and categorize security threats and attacks that in general, fall within those aspects in a systematic way.

### 5.1. Category

**Figure 2:**
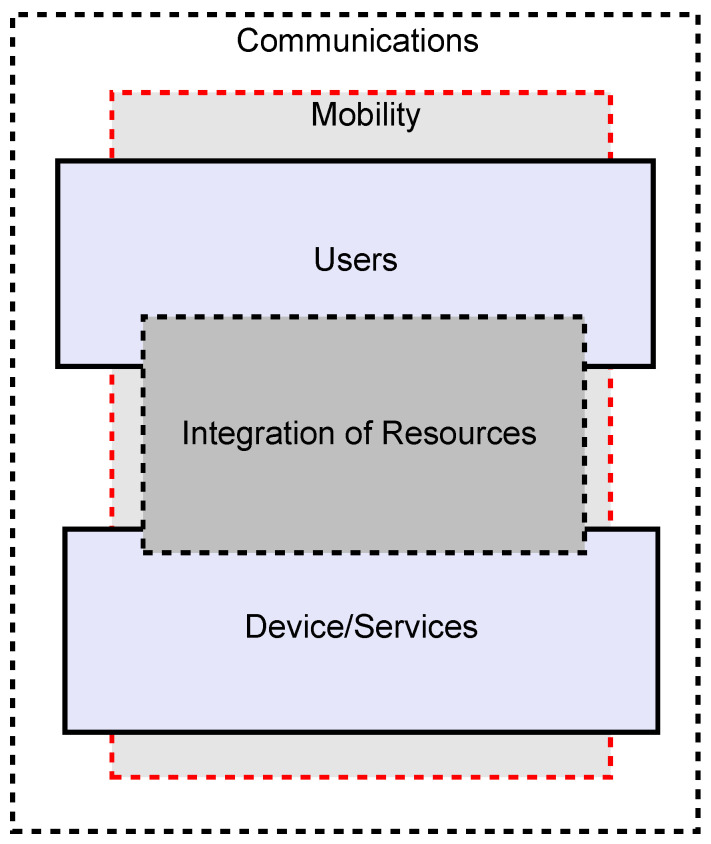
The devised threat and attack target categories.

In an IoT system, attacks may target a wide range of vulnerabilities [118,119,120,121,122]. These extend from the devices themselves, through the communication between them to the services and applications provided. Users and the inherently mobile and dynamic nature of these systems also provide attack opportunities. As well as considering the architectural characteristics of IoT systems in determining the security requirements and appropriate security architecture, the various possible threats and attacks on these systems need to be examined in arriving at an appropriate set of requirements and resulting security architecture. Based on the characteristics of the IoT, we categorize the possible threats and attacks into five areas. These are: *communications*, *device/services*, *users*, *mobility* and *integration of resources*. In Figure 2, we illustrate these categories. We use the term ‘services’ in a low-level sense, whereas the term ‘integration of resources’ covers applications that draw on multiple devices and services to meet end-user requirements. In this view, the IoT is comprised of communicating users and devices, the devices providing a range of services. Devices and their services are composed (service integration/division/composition) to meet end-user requirements. Both devices and users may be mobile (and dynamic). This view considers both the technical aspects of the definitions that are illustrated in Section 2, as well as the wider social, environmental, and end-users perspectives.

The category *Communications* covers the possible threats and attacks in wired and wireless medium (for instance, threats and attacks in routing channels and data transmission, etc.). *Device/services* encompasses physical IoT devices and their associated low-level services (for instance, related to battery, memory, data provision, etc.). *Users* covers threats and attacks (for instance, issues of data/location privacy and identity disclosures) on IoT users. *Mobility* is composed of the threats and attacks (for instance, that are related to the location-privacy and tracking, etc.) that exploit the movement of IoT *things* and smart devices. Finally, the *integration of resources* explores the threats and attacks (for instance, issues in cascading services/resources) that arise from the composition of diverse services into end-user applications. In Table 2, we present an outline of these categories.

The above categories take into consideration both the logical (e.g., edge intelligence, smart collaborations, service and integration, etc.) and technological (e.g., various processing and communication architectures, design methodologies, mobility, etc.) aspects of an IoT system. It is significant to note that a ‘communications’ attack may alter a packet with the intent of injecting malicious codes that will take control of a device. This example can be considered to be an attack on two different aspects of an IoT system - the communications and the devices. Similarly, many other such attacks involving multiple aspects exist. Next, we provide a detailed discussion of these categories.

#### 5.1.1. Communications

Communication lies at the heart of the IoT, with the connections between users and devices. Threats on this aspect of the IoT can be broadly grouped into categories e.g., *routing attacks*, *active data attacks*, *passive data attacks* and *flooding*.

In a routing attack, attackers target routing protocols and network traffic to either disrupt the flow of information or redirect the routing path to an insecure destination. They neither alter the contents nor attempt to gain information from the transmitted packets. Common forms of these attacks include blackhole, wormhole, and pharming [123]. The black-hole attack infects the network by preventing any network connection in the network. In this attack, the attacker node adapts the incoming data packets and drops the data packets while replying to the route request. In a wormhole attack, an attacker node entraps the data packets in one point of the network and tunnels them insecurely to one or more destinations without the knowledge of the sender of the data packets. In the pharming attack, the attacker node (in this case, a hacker) uses to steal the sensitive and private information of a victim over the Internet (i.e., making online fraud). Commonly, malicious codes are injected into the victim’s computer to compromised the computer system.

Active data attacks alter or delete information by targeting valid data packets directly rather than via subverting network routing. Examples of these attacks include channel jamming and various forms of data tampering (modification, manipulation, etc.) which may or may not result in valid packets. Active data attacks may target the payload, header or both of a packet. Passive data attacks attempt to gain information without altering the contents of communications. Examples here include eavesdropping and traffic analysis [30]. In an eavesdropping attack, the attacker node passively listens to information transmitted over the network between the sender and receiver. Similarly, in a traffic analysis attack, the attacker node intercepts and examines network traffic to determine the location, frequency of messages, analyze underlying payload traffic, and the identity of hosts.

Flooding attacks introduce new packets into the network. Examples of this include SYN flooding attacks (they are also regarded as a DoS attack). DoS attacks are of particular concern for IoT systems due to the resource-constrained nature of many IoT devices. It may only take a limited amount of bogus traffic before an IoT device is compromised by resource and bandwidth consumption [45]. Moreover, in a heterogeneous and decentralized IoT environment, the majority of IoT nodes (an IoT *thing*) perform networking functions by themselves in whatever wireless networking environment they belong to. In such non-trusted network environments, one common security issue can be the disclosure of private information to an unauthorized user by a packet dropping attack [56].

#### 5.1.2. Device/Services

Threats to the devices and services of an IoT system can be broadly categorized into *physical attacks*, *device subversion attack*, *device data access* and *device degradation*. The vast majority of IoT devices operate in open environments, where common security issues include device damage and disconnection. For instance, an attacker can physically disconnect an IoT device (e.g., a computer, mobile phone, even an air-conditioner) from the Internet, damage it beyond the point of serviceability or even destroy it completely [124].

In a device subversion attack (e.g., node or packet capture) an attacker assumes full or partial control over a device. This can then be used to actively cause the device to either cease functioning or to provide incorrect outputs. Taking control over IoT devices can be divided into two categories i.e., controlling a *single* device and controlling *many* devices. In the former case (i.e., controlling a single device), an attacker may, for example, penetrate a user’s home network (either physically or virtually) and take control of a single device (e.g., smart LEDs, refrigerator, etc). This can lead to its functionality being unavailable, or even restricted or misused. The low power of IoT devices make them more vulnerable due in part to the minimal (or non-existent) security protections that are embedded in such devices. Moreover, these devices are often incapable of updating to the latest software and security patches even when they have embedded security functionality. Importantly, we argue that these kinds of attacks are not unique to IoT devices, they are common for any networked computing devices. However, the constrained nature of IoT devices make them more vulnerable due in part to the minimal security protections that are embedded in those devices. Which may make it infeasible to update the software or patch to upgrade the latest security features against new threats and attacks [125]. In the latter case (i.e., controlling many devices), an attacker may assume control of many IoT devices (or many connected IoT devices) and manipulate services (*things* to human control), e.g., an attacker may disrupt a traffic monitoring service by controlling large numbers of the underlying sensors or attack the refrigerators in a retail store so that they will not cool their contents properly [126].

In a device data access attack, an attacker infects one or more IoT devices which are then used by attackers to perform malevolent activities on sensitive (and private) data without the user’s knowledge. It could happen using replay attacks or identity spoofing. For instance, stealing medical information by gaining unauthorized access to a patient’s mobile device (or any smart sensor attached to a patient’s body). Please note that the device appears to be functioning normally, but the data held by the device is available to the attacker [45].

Device degradation is a form of DoS attack intended to prevent access (by temporarily or indefinitely disruption) to a service by attacking the functioning of the devices themselves rather than the network’s ability to handle traffic. In a typical DoS attack the service is overwhelmed by having to process bogus traffic but the individual nodes are unharmed. However, in the case of the IoT this situation is more crucial. With their limited memory space and battery capacity, IoT devices can be attacked by memory exhaustion and battery corruption. Thus, a device degradation attack on these resource-constrained IoT devices in mass-scale can potentially unavailable resources and collapse the entire system’s operations [81].

#### 5.1.3. Users

We divide potential security threats associated with users into four broad categories i.e., *trust*, *data confidentiality*, *identity management* and *behavioural threats*.

With the potential scale of the IoT, trust is an even more pressing issue than is traditionally the case. Interactions may be fleeting and *things* will interact with a high number of previously unknown other *things*. Trust related attacks include self-promoting (in this case, a malicious node providing good recommendation for itself), bad mouthing (in this case, an attacker providing bad recommendation against a good node) and good mouthing (in this case, bad nodes providing good recommendations for other compromised nodes) attacks with the other peers located within the system [30].

The potential utility of the IoT lies in the richness of the data that it will contain. This may include extremely sensitive user data, e.g., age, address and medical data. A user’s privacy can be breached by any attack that accesses their personal information. Attackers may manipulate or disclose such data or use it to impersonate the user (i.e., user impersonation attacks) [56]. User impersonation in the IoT is a critical issue due to the combination of heterogeneous data sources coming from various IoT *things*, contexts and locations. This can be done via identity spoofing, where attackers gain unauthorized access to IoT systems. One way to obtain a user’s confidential information is via a phishing attack, in which attackers steal valuable and confidential personal details e.g., user-name and password or credit card number. Others include attacks on anonymity supporting protocols [127].

With the IoT’s scale and heterogeneity and an expected user desire for privacy, it is likely that users will maintain multiple identities [45]. This also multiplies the normal vulnerabilities that attackers can exploit, due to the range of interactions of the systems supporting these identities. In IoT systems, management of identities is a major concern for authenticating and authorizing a legitimate *thing* (e.g., who and what is connecting to), where the service provider and the service consumer may both try to keep their identities hidden. Attackers may exploit the heterogeneous and multi-domain nature of the systems supporting identity management in the IoT to subvert these systems. In personal and social domains (includes social networking), users’ malicious or selfish behaviours can also be used to create attacks through social engineering. For example, by downloading malicious software or being tricked into revealing private information through phishing attacks, or making a free-rider attack in which the attacher (named a free rider) takes benefit from the community without contributing their share to the community [123].

#### 5.1.4. Mobility

We divide the various mobility related security issues into three categories i.e., *dynamic topology/infrastructure*, *tracking and location privacy* and *multiple jurisdictions*. As noted above, some threats can be viewed from multiple perspectives, for example users’ mobility may increase the possibility of active and passive data attacks (communications) and location tracking (mobility).

In the IoT, complex network structure and the characteristics of the system itself present challenges e.g., changing topology and flow. Due to such a dynamic topology and the resource constrained nature of the IoT devices, the routing for transmitting data becomes crucial [128]. Commonly, in the IoT, nodes do not necessarily need to connect over the Internet, but they can connect via any network e.g., WSN, WLAN or Personal Area Network (PAN). In such an environment, when users and devices move (i.e., joining and leaving the network), the network topology is dynamically modified. This could generate security challenges of interdependencies (e.g., attacks on networked-car, electronic medical devices and power stations) for the end-users. This could further evolve into ‘sinkhole’ attacks by attackers altering the network topology (by advertise its fake routing updates) and traffic flow, and gaining illegal access to a user’s data in a real-time situation [30,129].

Smart IoT devices connected to the Internet could disclose a user’s geographical location through time and space [130]. The location-based services can be categorized into two types, namely location tracking and position aware services [131]. In tracking and location privacy, information (e.g., user’s current position, daily routine or certain activity) in an IoT system could be inherently vulnerable and a possible point for attackers to target to breach personal privacy. On the other hand, position-aware services generate vulnerabilities based on the device’s own knowledge of its position [132]. Thus, information related to a user’s physical location and activities can bring considerable privacy risks for both the users and the systems.

It may be also possible that several disjointed networks of *things* join to form inter-domain collaborations and co-ordinations. It is likely that such collaborations will use heterogeneous technology. Attackers may seek to exploit any mismatch in policy settings, identity management or security technologies. For instance, in a traffic accident police officers can communicate with emergency services to coordinate the well-being of the driver or passengers. However, the management of this information over the jurisdictions possess several challenges (both technical and legal) of data privacy due to the regulations in different jurisdictions [133].

#### 5.1.5. Integration of Resources

In the IoT, from data collection to data processing, storage and usage are highly dependent on diverse infrastructures in terms of reliability, scalability and security [134,135]. The data from individual devices, possibly in very large numbers, are aggregated to provide integrated services and applications to the end users. The components which co-operate and interact to provide end-user results may be controlled by multiple different domains. Even when control resides within a single domain, there are challenges in ensuring security at each stage of the composition. We divide the threats in this area into three categories i.e., *cross domain administration*, *cascading resources* and *interoperability*.

IoT systems may involve components from many different network domains. It was reported that according to the surveys of 439 million household’s network usage of WiFi network connections, 49% of WiFi networks are insecure and 80% of households use their default network passwords. Additionally, it was observed that 89% of the public hotspots are insecure due to the lack of a trusted network connection [136].

Moreover, in a decentralized IoT environment the majority of the IoT nodes perform networking functions by themselves in whatever wireless networking environment they belong to [137]. Here again, attackers may seek to exploit any mismatch in policy settings, identity management or security technologies.

End-user applications in the IoT can potentially draw upon a vast range of *things* and services. Any security breach at the low-level may cascade up and affect higher level services and applications that depend on the compromised component. For instance, an attacker can penetrate a user’s mobile network and make a modification to their home automation system and compromise a motion sensor. If the system is set to open windows or doors when motion is detected the attacker may be able to gain access to the building [114]. As another example, an attacker may introduce malicious code into a poorly protected device (i.e., poorly secured). The code is then passed up as data through applications and used to infect user devices by compromising its functionality. Furthermore, the large volume of data in the system can create threats to the user’s privacy and information security. In such attacks, the attacker gathers a large amount of information (of service, user and resources) and may perform automated data-mining without being noticed by the user and service provider [116].

Interoperability relates to attacks based on the need for multiple systems collaboratively to work together and the ability of attackers to exploit any potential issues in an IoT system. Such systems can consist of a combination of cloud computing, fog computing, social networks, mobile computing and industrial networks [134,138]. The security settings and policies of such systems may not easily integrate, leaving vulnerabilities as data is moved and communicated between components. For instance, in a smart healthcare system, a patient’s data (e.g., blood pressure, blood glucose level, etc.) is collected, analysed and transferred to the patients by the doctors, which may depend upon several of these dynamic networks and components. Therefore, at any of these stages an attacker can breach a patient’s private and sensative information by penetrating the networks between the infrastructures [139].

In Table 3, we precisely illustrate these various threats and attacks categories and related mechanisms discussed above (i.e., in Section 5.1).

## 6. IoT Security Requirements: State of the Art

In this section, we provide some related work in the state of the art security requirements in the IoT. We take an analytical approach to the literature in order to examine and explain different security requirements, specifically why or how it is a requirement. The articles examined address a variety of concerns, for instance, the IoT and its general security issues, IoT security requirements, specific security problems and approaches within the IoT and IoT security architectures and their assessment. We observe that there have been a limited amount of previous work that specifically addressed security requirements for IoT as a central issue, with other work providing analysis of IoT security needs and architectures and security requirements treated as secondary issue. As noted by Alqassem [140], providing specific security requirements for the IoT is both necessary and difficult.

Yang and Fang [141] presented a security architecture for the IoT based on communication, control and computation aspects and discuss the basic IoT security issues e.g., authentication, access control and identity. However, how this architecture would address the characteristics of devices in the context of an IoT system (and the security of IoT services and applications) and exactly what requirements are being addressed, is unclear. The answers to questions about users’ interactions and systems scale are also missing.

Alfaqih and Al-Muhtadi [142], and Sain et al. [143] discussed IoT security requirements based on the different architectural layers (e.g., physical, network, application layers etc.) and examine communications between them. The critical issues e.g., fault tolerance, authentication, access control, privacy, and confidentially are discussed. However, how to combine these requirements for creating a secure IoT system is not addressed in this literature.

In [144], Isa et al. presented a security architecture for the IoT. The architecture considers the issues of secure protocol for transferring a high volume of data between embedded devices. The proposed architecture is composed of four layers, namely hardware, firmware, operating system, and application. However, the proposal is limited to discuss the proposed protocol and no attempt is made to relate this architecture to examine wider IoT security requirements.

Heer et al. [145] presented an IoT architecture and discuss the security needs of such an architecture considering the viewpoint of device life-cycle. The proposal considered an in-depth analysis, regarding the issues e.g., scale, heterogeneity, end-to-end security, and issues of centralized versus distributed architectures. However, the discussion is limited to the existing Internet protocols.

Several authors presented general security analyses of the IoT. For instance, Li et al. [44] gave an IoT security analysis based on a four-layer model of the IoT. These layers are sensing, network, service and application-interface layers. While the authors address the issues of security, privacy and usability within the IoT, they do not directly address the issue of scalability in the number of *things* within the system and do not propose any actual design. A similarly layered, but very different, architecture for the IoT was presented by Misra et al. [146]. This architecture consists of four layers. (i.e., *things*, network, management and analytics). The architecture addresses the interaction between different physical and virtual *things*, sensors and network services, data integration and management, and analytics of the collected data.

Roman et al. [45] presented a view of IoT security, that includes edge intelligence, resource-constrained service provisions (at the edge of the network) and collaborations. Several approaches to IoT system design are discussed, including centralized, collaborative and distributed IoT. Features and security issues of distributed IoT are analysed in depth, but the paper does not provide any systematic analysis of security requirements for the IoT.

Abomhara et al. [147] further considered the issues discussed in [45], and explore the requirements of an IoT security architecture from the point of three core issues i.e., privacy for humans, confidentiality of business process and third-party dependability. While the paper recommends employing cryptographic techniques and light-weight security mechanisms into the *things* located at the edge of the network and takes a useful application-based perspective, its final recommendations go little beyond traditional security issues of authorization, authentication, identity management, trust and key management mechanisms.

Sicari et al. [30] proposed the construction of a scalable and structured IoT security architecture and propose security requirements e.g., integrity, confidentiality, authentication, privacy, trust and mobility. Apart from the conventional security requirements, this paper also examines the requirements for an end-to-end security integration and verification in IoT architectures. Unlike [44], this paper focuses on the IoT security requirements from the logical (e.g., *things* interactions) and technological (e.g., *things* collaborations) points of view.

Unlike [30], Kim et al. [148] discussed IoT security requirements based on the IoT gateway systems considering the scale of the IoT in an open wireless network environment. The major issue they mention is the application of efficient light-weight cryptosystem for information security. However, they do not discuss the critical issues e.g., interoperability, cross-domain network management and communication security over multiple jurisdictions.

Singh et al. [149] went beyond the requirements proposed in [148], which focuses on a particular security domain, and explore security requirements for cloud-supported IoT systems. They mainly focused on the security provisions from the perspective of cloud-tenants, end-users and cloud providers while integrating them in the IoT. The significant issues of wide-scale, cross domain platform and the case of multiple jurisdictions have been taken into consideration. The paper also presents a detailed discussion on the scalability, access control, identity encryption, trust, location privacy both for the users and the *things*, composite service management supporting a decentralized network architecture. Likewise [148], this paper emphasizes the importance of the light-weight key management systems for IoT devices, given their limited battery and processing capability.

Similar to [149], Zhou et al. [150] discuss security and privacy issues for the cloud-assisted IoT systems. However, unlink [149], this paper is limited to the security issues in the context of secure packet forwarding.

Babar et al. [151] discussed a threat taxonomy for the IoT and propose high-level of security requirements. Apart from mobility, scalability and access control, they explored diversity in the computational abilities of the IoT devices. The authors argued that IoT systems should be resilient to attacks, i.e., the system should avoid single points of failure. They also mentioned the need for data authentication, client’s privacy, secure storage and communication and tamper resistance (i.e., maintain the device security even if the device is accessed by the malicious users).

Al-Fuqaha et al. [56] presented an overview of IoT security in regards to enabling technologies, protocols, services and applications. The paper outlines the security challenges including availability, reliability, performance, mobility, scalability, interoperability and trust. It also provides an overview of security requirements for the IoT based on different architectural layers e.g., physical, network, application, etc. In a similar way of [56], Gluhak et al. [152] discussed the security requirements for the IoT based on scalability, heterogeneity, reputability, federation (i.e., one security domain can access resources on another security domain), concurrency and mobility. However, unlike [56], this paper focuses the security requirements in the context of experimental IoT research (i.e., evaluation based on the realistic conditions in real-world experimental deployments).

Similar to [152], Huang et al. [153] discussed security issues for the IoT and outline the security requirements based on real-world experiments that investigate three different IoT scenarios, namely body IoT (e.g., wearable IoT-enabled healthcare devices), home IoT (e.g., intelligent sensor for tuning on and off lights) and hotel IoT (e.g., IoT enabled smart hotspots). However, their approach to security requirements is very low level and context dependent, for example including issues e.g., ‘door access control system’ and ‘hotel payment’. This makes their approach difficult to generalize to analyse IoT security requirements.

Yaqoob et al. [154] presented a taxonomy for the IoT, including architectures, topologies and enabling technologies. They also present a four layer architecture, with security located in one of the four layers (management and security services). They discuss several requirements for an IoT architecture e.g., resource control, energy awareness, interoperability, QoS, interference management and security. They also list some of these as open challenges and also include the other issues e.g., scalability, flexibility and mobility. While they do not give precise security requirements, their more general approach can contribute to the discussion. They further emphasise that the security requirements must support remote resource management and proper resource use.

Alam et al. [155] discussed interoperability issues between different administrative domains for the IoT. They list standard security requirements: confidentiality, integrity, availability, authentication, authorization, access control, trustworthiness, and auditing. While they presented a practical reference architecture and a real-world experimental test-bed, they do not present IoT specific security requirements. In particular, their discussion gives very little emphasis to scalability.

Cirani et al. [156] examined security needs for the IoT within the context of the IP (Internet Protocol). They analysed existing Internet protocols used in the IoT to arrive at security challenges and requirements. Their main conclusion was around the need for light-weight protocols to deal with the scale of the IoT and the nature of IoT *things*. While we agree with this conclusion, [156] does not offer any more in the way of IoT specific security requirements, instead relying on basic security properties of confidentiality, availability, integrity and authentication.

Several other proposals also outline the importance of IoT security and discuss various security requirements. For instance, Hossain et al. [157], and Park and Shin [158] listed IoT security requirements that include data integrity, information protection, anonymity, non-repudiation and data freshness (i.e., real-time data). Alqassem [140] discusses IoT security requirements from a heterogeneous network’s perspective, here the author focuses on secure and private connections and transactions.

Other contributions discuss IoT security requirements based on large-scale applications, specific to technological consequences of the IoT. For instance, Schaumont [159], and Jaiswal and Gupta [160] discuss security issues aligned to IoT-enabled healthcare systems, and outline the challenges and security requirements. Along with the traditional security requirements of access control, authentication, authorization, the authors argue the need for self-healing, trust, fault tolerance and light-weight key management protocols.

Many other existing approaches (e.g., [161,162,163,164]) pose authorization, authentication, confidentiality, access control, trust and identity management as the core security requirements for an IoT system. In addition to these requirements, a few studies (e.g., [165,166,167,168,169,170]) discuss other general security requirements e.g., network security, application security, layer security, bootstrapping security, configuration, data integrity, firewalls, anti-virus and encryption functionality and secure routing. We consider these generic as such requirements are necessary for most, if not all, application areas.

In Table 4, we summarize the potential security requirements for an IoT system that are discussed in the aforementioned proposals. The requirements highlighted in ‘red colour’ can be regarded as ‘standard’ security requirements.

## 7. IoT Security Requirements: Our Approach

The goal of this section is to examine our approach to IoT security requirements. As can be seen from the analysis presented in the previous section and collected in Table 4, a large range of requirements for securing the IoT has been presented in the literature. Many of these requirements (highlighted in ‘red colour’ in Table 4) are familiar from other application areas and can be regarded as ‘standard’ security requirements that need to be met by any secure system. These include confidentiality, access control, authentication, authorization, availability, key management, integrity, trust, non-repudiation, accountability and usability. That is, any security system, not just the IoT, should be designed with due regard to these requirements. Obviously, security architectures, systems and mechanisms for the IoT also needs to adhere to these requirements.

What can also be observed from Table 4 is that several security requirements, much more specific to the IoT context, have been proposed. It can further be seen that these requirements can overlap and and/or require tradeoffs. In this case, the right balance between the *primary* requirements must be achieved. In Table 5, we propose a more succinct set of requirements, based on the entries in Table 4. These requirements are discussed below.

- *Light-Weight Solutions:* Any proposal for IoT security should consider the resource-constrained nature of the *things* and support light-weight solutions. Constrained resources include computational limitation which puts some limit on the implementation of cryptographic techniques and protocols supported by the device. This requirement also addresses the issue of energy awareness identified in Table 4. It expands upon energy usage and power generation with respect to the computing processing power of the constrained IoT devices. In other words, light-weight security solutions must achieve a balance between the employed cryptographic techniques and improved energy aware communication by optimizing energy consumptions. Many authors have mentioned the need for light-weight solutions in the IoT, but it has rarely been expressed as an explicit security requirement.
- *Decentralized Management:* The IoT is potentially a system of huge scale. Centralized solutions are unlikely to be generally practical. Proposals for IoT security must allow for a flexible approach to placement of security provisioning and management to address the issues around centralized versus decentralized architectures. Given the edge intelligence present in many IoT systems and their potentially large-scale, the security provisioning should be placed as close as possible to the point of need, while allowing for resource-constrained devices. This will likely take the form of decentralized management responsible for clustered portions of the system. This requirement addresses the issues of manageability and decentralization identified in Table 4.
- *End-to-End Security:* The IoT will not only be large but heterogeneous, with communications passing through multiple administrative domains and across multiple technologies. Security provisioning must encompass the entire scope of a connection. This requirement covers the issues of secure storage, secure communication, secure content (i.e., information typicality protected from unwanted users and applications) and QoS from Table 4. This will require interoperable security technologies, inter-domain policy management between the end points and identities that are verifiable between the end points. Network QoS can be seen as a key issue for securing networking in electronics communication. It is important to protect crucial QoS parameters during communication setup and the protection of data packets during their transmission.
- *Identity Management:* In a large scale, highly dynamic and decentralized, system, e.g., the IoT, issues of identity and anonymity, whether at the individual device or higher grouping level, will be extremely important. IoT security must provide reliable techniques for administering the identities of devices and users and for flexibly handling relationships between these identities. This will include providing for seamless integration of various services connecting different devices and users across multiple domains, flexible support for identity management and mutual authentication for users, devices, applications and associated services. Security solutions must recognise that in general, it will not always be possible to have foreknowledge of the participants in an interaction and provide mechanisms to deal with the scale of the number of identities in the IoT. This issue of scale will also mean that identity cannot always be handled in a fine-grained manner and identities will often have to be handled in more scalable manner, e.g., by using one identity to refer to multiple entities. While identity may sometimes be considered a general security requirement, the scale of the number of *things* means that the IoT will require innovative approaches to identity management. This requirement covers the issues of identity and anonymity identified in Table 4.
- *Privacy:* Just as with identity, the scale and nature of the IoT requires a particular focus on issues of privacy, in all its forms. Users will wish to maintain their privacy and quickly and precisely obtain the services they require. This requirement covers the issues of privacy and location privacy identified in Table 4.
- *Mobility/Dynamic:* As already noted the IoT has the potential to be of extremely large scale and its individual components may be highly mobile. This will make such systems highly dynamic. The scale of the variations in structure, location and architecture must be taken account of in any security proposal. The mobility of connected devices, users and *things* must be supported by security solutions that ensure smooth transition of jurisdictions and information sharing between various devices, users and *things* smoothly. Such solutions also need to support mobile access to data and applications leveraging system dynamics and location-aware services. This directly addresses the issue of mobility identified in Table 4.
- *Scalability/Incremental Deployment:* One important consequence of the scale and heterogeneity of IoT systems, in general, is that such systems will continue to grow and be deployed after operation commences. Significant revisions, adaptations and realignment will occur, while the system continues to operate. Security must be provided while this is occurring, and the system scales up. This is in contrast to many systems, where the security is designed with the assumption that the system will be deployed and then used. Security solutions for the IoT must support this style of deployment, allowing *things* and users to join and leave the system and system functionality to dynamically evolve. In concert with this security solutions must be scalable, to meet the potential size of the IoT. This requirement addresses the issues of scalability and load balancing identified in Table 4.
- *Robust and Reliable:* IoT systems need to be robust due to such factors as mobility, device faults and the increase in the number of attack vectors. Security solutions for these systems will need to demonstrate the same characteristics. The solutions should support self-repair. This means that security systems should have the ability to discover faults and correct them by taking appropriate measures automatically. IoT security must support, and make use of, real-time data analytics, aggregation and their efficient usage that are collected by the smart sensors. There must also be support for data freshness by ensuring that the data is current and immediately usable and useful. Data may only be available for a small-time window, as in when real-time data is collected. Any security solution must provide for integrity between various functional components and their services in the large-scale IoT applications commerce e.g., smart home, smart city, smart transport, smart health, etc. Due to the intensely dynamic nature that the IoT may display, security provision must be able to react in real time to address any threat. This group of requirements cover the issues of real-time, reliability, data freshness, resilience to attacks and fault tolerance identified in Table 4.

While the above discussed requirements represent a synthesis of the existing requirements for IoT security as stated in the literature, we do not consider this completely accounts for all the security needs of these systems. From the analysis in Section 6, it can be seen that the IoT has certain features that set it apart from other systems. For example, it is extremely large, may consist of an extremely diverse range of hardware and software, which must communicate and co-operate across multiple, independent, administrative domains. While these can be seen as aspects of scalability, not all scalable systems need to deal with these issues. They may have fixed hardware/software components and be under a single administrative domain. This does not apply in the IoT. In addition, to their own needs, the scale and mobility aspects of the IoT may lead to new types of interaction, e.g., transient/ephemeral relationship between component entities, where they only interact for a limited lifespan/number of times. Perhaps only once within their active lifetime. This leads to several requirements not mentioned previously in the literature (we highlighted them in ‘X’ and ‘red colour’ in Table 6). These requirements are discussed below.

- *Composition/Heterogeneity/Interoperability:* The IoT composes and aggregates services and data from devices to provide diverse applications to users. Appropriate security must be provided at each stage of this composition and should be preserved by the system as a whole. Security solutions must account for the heterogeneous nature of the IoT in terms of devices, technologies, etc. The security design and implementation should not depend upon a specific technology, rather it should be technology agnostic. Furthermore, given the dynamics in the IoT networks and importantly the potential heterogeneity in the devices, the issue of interoperability must be considered when providing security for the IoT.
- *Transiency/Ephemeral:* Relationships in the IoT systems consisting of a multitude of users, each possessing a large range of smart devices, may be fleeting, with users, devices and systems that have never encountered each other before interacting, possibly on a one-time-only basis. IoT security solutions must support such interaction modes knowing the present network congestion to avoid an unnecessary overhead.
- *Federation in Administration Domains:* In an IoT system, it may be possible that several disjoint networks of *things* make inter-domain collaborations and coordination within a jurisdiction or over several jurisdictions. The data and services of the IoT may be offered by multiple, co-operating domains. Some services may be composed of elements that are controlled by different authorities, and processes may require data to pass across domain boundaries. Any security solution must enable the relevant security policies of all involved domains to be enforced and administered.

## 8. Discussion and Lessons Learned

In this section, we summarize the lessons learned from the aforementioned analysis of requirements for IoT security. Most of the work discussed in Section 6 identified the necessity for IoT security requirements by addressing generic or standard security needs. These are the common requirements for providing security for any computing system, and obviously need to be addressed within the specific context of the IoT. In addition, there are other requirements specific to the IoT, as discussed in Section 7. Please note that not all requirements will apply in the same way in all aspects of an IoT system. We have also observed that threats and attacks can occur at multiple points within an IoT system both in terms of components (the physical *things* that make up the system) and logically (at the various points of the system architecture).

We noted that the previous proposals identified various requirements for the provision of an IoT security architecture. For example, Isa et al. [144] and Babar et al. [151] mention physical attacks on devices. The issues of heterogeneity and scale of IoT systems are mentioned by several proposals (including [44,45,146,148,149]). Proposals, for instance, Abomhara et al. [147] and Gluhak et al. [152] identify the need for light-weight security solutions for the resource-constrained IoT devices, an issue also noted by Heer et al. [145]. The issues of centralized versus distributed approaches, are considered by several authors (e.g., [30,45,145,146,147,171]). It can be seen that there are trade-offs involved in the question of centralized versus distributed approaches. On the one hand, too much decentralization risks a loss of control and vulnerabilities occurring in the independent components. On the other hand, too much centralization risks the creation of unscalable solutions. Several proposals discuss the scalability issue of the IoT in terms of the devices and users. However, a few of them examine the scalability issue in terms of the differing needs that is composed of services and applications. While Li et al. [44] propose a layered security model for the IoT, the distribution of functions to layers resembles inconsistent, for instance, user authentication is retailed only at the service layer and not the application layer.

Based on the forgoing analysis, we have introduced a set of security requirements for an IoT security architecture. We observed that some of these requirements can be employed as an individual requirement and some others can relate to each other to be grouped together to present a more manageable set of requirements. For instance, identity management, end-to-end security and decentralized management can be seen as an individual requirement, whereas, robustness/self-healing/reliability/real-time/data-freshness can be grouped together to present a manageable set of requirements. Once again, it is worth mentioning that the generic or standard security functionality will need to be provided throughout the architecture potentially at difference levels of granularity.

Recall, in Section 3, we have illustrated different layers of an IoT security architecture (shown in Figure 1) and outlined some common security issues for each layer. Here, at first we briefly recall some notable security issues in each layer of the IoT security architecture in the light of our discussion presented in Section 5. Then we consider each layer of the architecture and show how our proposed requirements apply.

### 8.1. Security Requirements for the Device Sensing Layer

The common security issues in the device sensing layer are authentication, authorization, access control and identity management for the devices. Among others, physical attacks, device subversion attack, device degradation and devices’ data access are major threats and attacks for this layer. Important security requirements in this layer are light-weight security solutions, support for composition/heterogeneity/interoperability and robustness/self healing/reliability/real time/data freshness. Recall, the security design must be light-weight in order to make their compatibility within the constrained IoT devices. Furthermore, given the constrained nature of the IoT devices, the security provision could be arranged in a decentralized way, placing them as close as to the devices themselves. Additionally, the design must support efficient identity management for allowing access against legitimate devices for an authorized service, partially rely on underlying infrastructures. The support for mobility/dynamic in this layer is crucial, as the devices can move from one location to another frequently within their life-cycle. The security proposals must also support the scalability/incremental deployment, as the devices can be dynamically added to the system at scale.

### 8.2. Security Requirements for the Network Management Layer

Among others, issues e.g., routing attack, active data attack, passive data attack, flooding, dynamic topology/infrastructure, cross domain administration and multiple jurisdictions are major pitfalls in this layer. Major security requirements for the network management layer are the support for composition/heterogeneity/interoperability, light-weight security solutions for communications, scalability/incremental deployment in technologies and networks, the federation of administration domains and support for mobility/dynamic. The employed security mechanisms could be placed in heterogeneous network infrastructures e.g., cloud or physical servers. These mechanisms must be scalable and ensure communication security and privacy leakage by fortifying data integrity during the life-span of the network. The federation of administration domains must be considered for managing networks in different authorities and multiple domains. The design, in addition, need to support the mobility/dynamic in communications and routings within a certain network or between multiple networks.

### 8.3. Security Requirements for the Service Composition Layer

Notably, flooding, devices’ data access, data confidentiality, cascading resources and interoperability are significant security issues in this layer. Major security requirements in this layer are robustness/self healing/reliability/real time/data freshness, scalability/incremental deployment in the number of services, composition/heterogeneity/interoperability in service management. These requirements would help to satisfy the common need of trustworthy data management that must capable of doing event processing, service division/integration, service decision, service monitoring, service configuration and performing various decision analytics according to the policy requirements and contextual information. Other essential requirements for this layer are the support for decentralized service management. Supporting decentralized management, security provisioning could be placed locally (i.e., within the IoT devices) or these could be placed in a decentralized data repository based on the system’s requirement. In addition, the design must support transiency/ephemeral to support interactions among the services without an unnecessary overhead.

### 8.4. Security Requirements for the Application Layer

Threats and attacks e.g., cascading resources, interoperability and multiple jurisdictions are major concerns in this layer. Significant security requirements for the application layer are robustness/self healing/reliability/real time/data freshness in data presentation, composition/heterogeneity/interoperability in application maintenance, light-weight security solutions of access control and end-to-end security provisioning for the applications. Most of the existing applications come with the distinct apps that contain service-specific functionality to provide essential smart services and communication interfaces directly to the end-users. However, these applications are more likely to be vulnerable to possible attacks. In addition, the design should scale the number of applications which shows the ability to support an increasing number of applications without any degradation in the QoS. The security design needs to support privacy (both data and location) when dealing with various applications that evolve and transform in response to threats.

### 8.5. Security Requirements for the User Interface Layer

Common security issues, among others, in the user interface layer include authorization, authentication, access control and identity management for the users. Importantly, trust, data confidentiality, behavioural threats and tracking and location privacy are some major concerns in this layer. Major security requirements for the user interface layer are end-to-end security, support for mobility/dynamic, scalability/incremental deployment, composition/heterogeneity/interoperability and decentralized management. The employed security design in this layer must follow the adaptation of encryption protocols for ensuring end-to-end secure communication between the users. These security provisioning could be placed inside a user’s device or within some local operators controlling the devices through authorized remote maintenance supporting the decentralized service management. The security design also need to support the scale in the number of users within the system to offer efficient services. Nevertheless, it is also necessary to employ flexible identity management mechanisms to protect user’s privacy.

In Table 7, we outline some notable security requirements in different layers of the IoT security architecture that we illustrated in Figure 1.

## 9. Conclusions

The IoT is a trending technological field that provides a platform to make several opportunities with a range of cost effective, efficient and ease of use applications and services to the end users. However, security is one of the pressing issues towards a wider deployment of the IoT systems. In this paper, we have identified and examined the state of the art security requirements in the IoT and proposed a set of security requirements for an IoT security architecture. We presented a detailed discussion on the issues that need to be considered for building such a secure IoT architecture based on the proposed requirements. We have observed that the current security requirements for the IoT are not structured to handle the various threats and attacks in an organized way. We noted that there is a need for a systematic approach for addressing IoT security requirements that is able to capture the basic needs to build a secure IoT architecture.

Unlike the traditional IoT security analysis, we have classified the various security issues into five distinct categories, namely threats and attacks in communications, device/services, users, mobility and integration of resources, and examined potential threats and attacks for each of them. Based on these categories and available solutions, we have devised the list of security requirements that are pivotal for securing an IoT system. Please note that our proposed requirements are not the unique and mandatory for IoT; however, by following them it could be easier to achieve the much of the promised benefits of scalability, usability, reliability, trust, identity, etc. It is noted that without a proper assessment of possible threats and incorporation of appropriate security requirements, an IoT system will be vulnerable to attacks. We have examined and detailed the various security issues in different layers of an IoT security architecture. We outlined these various critical security requirements in five distinct layers of an IoT security architecture, namely devise sensing layer, network management layer, service composition layer, application layer, and user interface layer. We observed that there may be an overlapping of certain security requirements in two different layers. However, they are dependent upon the system’s requirements and the designer’s choice. In future, we plan to conduct empirical studies with the proposed security requirements to assess security issues in more detail for a practical IoT system. We also plan to perform a comprehensive experiment with different use-case scenarios which will help to derive further insights to constitute a secure IoT system in a real-world scenario. 

## Figures and Tables

**Table 1:** The core components, major functionalities and common security issues for each layer of the IoT security architecture depicted in Figure 1.

Architectural Layers	Core Components	Major Functionalities	Common Security Issues
*Device sensing layer*	Smart sensors, actuators, RFID tags	Data sensing, data acquisition	Authentication, authorization and access control.
*Network management layer*	Wired, wireless networks, big data repositories	Data aggregation, QoS scheduling	Unauthorized access, modification of routing paths.
*Service composition layer*	Middle-ware technology, heterogeneous objects	Analysis and processing of data	Service (or group) authentication, data confidentiality.
*Application layer*	Various applications e.g., smart home, smart city	Determines massage passing protocols	Unauthorized access, privacy leakage, integrity.
*User interface layer*	Users	Export services to the end-users	Authentication and authorization, data confidentiality.

**Table 2:** An outline of the devised threats and attacks categories and their brief description.

Category	Brief Description of Threats and Attacks
*Communications*	Threats and attacks in wired or wireless mediums e.g., routing channels and data transmission.
*Device/Services*	Threat and attacks for the physical IoT devices and associated low-level services e.g., battery.
*Users*	Threats and attacks of the human being in an IoT system e.g., privacy and identity disclosure.
*Mobility*	Threats and attacks exist in different network domains e.g., location-privacy and tracking.
*Integration of resources*	Threats and attacks exist in a heterogeneous infrastructure e.g., cascading services/resources.

**Table 3:** Devised threats and attacks categories and related security mechanisms.

Security Categories	Security Issues	Threats and Attacks
*Communications*	*Routing attack*	*Blackhole*
		*Wormhole*
		*Pharming*
	*Active attack*	*Jamming channel*
	*Passive attack*	*Eavesdropping*
		*Traffic analysis*
	*Flooding*	*DoS/DDoS*
		*SYN Flooding*
		*Routing table overflow*
*Device/Services*	*Physical attack*	*Device disconnected or damage*
	*Device subversion*	*Device control/capture*
	*Device data access*	*Replay attack*
		*Identity spoofing*
	*Device degradation*	*State manipulation*
		*Battery exhaustion*
		*Heat stroke attack*
		*DoS/DDoS*
*Users*	*Trust*	*Self promoting*
		*Bad mounting*
		*Good mounting*
	*Data confidentiality*	*User impersonation*
		*Identity spoofing*
		*Phishing*
	*Identity management*	*Subversion attacks*
	*Behavioural threats*	*Malicious users*
		*Social engineering*
		*Free riding attack*
*Mobility*	*Dynamic topology/infrastructure*	*Trust related attacks*
		*Network/device related attacks*
	*Tracking and location privacy*	*Device tracking*
		*Tag tracking*
	*Multiple jurisdictions*	*Attacks on policy settings*
		*Data privacy*
*Integration of resources*	*Cross domain administration*	*Attacks on policy settings*
		*Identity management*
	*Cascading resources*	*Malicious node manipulation*
		*User’s privacy*
		*Information security*
	*Interoperability*	*Data privacy*

**Table 4:** Potential security requirements for the IoT identified in the existing literature. Red colour represents ‘standard’ security requirements.

Requirements	[152]	[151]	[148]	[149]	[147]	[154]	[145]	[155]	[156]	[168]	[169]	[153]	[143]	[158]	[157]	[140]	[160]	[170]
Confidentiality				✓					✓	✓	✓	✓	✓	✓		✓	✓	✓
Access control	✓	✓	✓	✓	✓	✓	✓				✓		✓	✓	✓			
Authentication		✓		✓				✓	✓		✓		✓		✓	✓	✓	✓
Authorization		✓		✓				✓	✓					✓			✓	
Availability		✓	✓						✓			✓		✓				
Key management	✓		✓		✓		✓		✓									
Integrity				✓				✓			✓	✓	✓	✓	✓		✓	✓
Trust	✓			✓				✓			✓			✓			✓	✓
Non-repudiation											✓			✓	✓		✓	
Accountability											✓			✓		✓		
Usability							✓									✓		✓
Scalability	✓	✓	✓	✓	✓	✓									✓	✓	✓	
Resilience to attacks		✓		✓				✓							✓		✓	
Privacy		✓			✓					✓	✓						✓	✓
Identity		✓		✓	✓		✓		✓	✓	✓			✓			✓	✓
Secure storage		✓		✓	✓													
Secure communication				✓	✓				✓							✓	✓	✓
Secure content									✓	✓	✓							
Manageability			✓			✓												
Location privacy						✓					✓			✓				
Decentralization	✓			✓	✓											✓		✓
Energy awareness						✓											✓	
Quality of service				✓		✓												✓
Anonymity									✓	✓	✓			✓	✓		✓	
Real-time		✓													✓	✓	✓	
Data freshness						✓		✓								✓	✓	
Reliability	✓		✓									✓				✓		✓
Load balancing				✓					✓		✓							
Mobility	✓		✓			✓							✓					
Fault tolerance						✓							✓			✓		

**Table 5:** The list and a brief outline of our approach to the IoT security requirements.

Proposed Security Requirements	Brief Description
*Light-weight solutions*	Consider resource-constrained nature of the *things* (not just energy but computer power).
*Decentralized management*	Support for a flexible security provisioning in a decentralized nature.
*End-to-end security*	Support end-to-end security where the communication path may traverse multiple domains.
*Identity management*	Incorporate flexible and reliable identity management techniques for registering *things*.
*Privacy*	Consider privacy of users and data.
*Mobility/Dynamic*	Support the mobility of devices, users and *things*.
*Scalability/Incremental deployment*	Support the large volume of data, applications, services and users.
*Robustness/Self-healing/Reliability/Real-time/Data-freshness*	Support security across a wide range of operational conditions, discover the faults and correct them automatically.
*Composition/Heterogeneity/Interoperability*	Consider and support the diverse security requirements for different types of services and applications.
*Transiency/Ephemeral*	Support a short interaction (between the *things*) without an unnecessary overhead.
*Federation in administration domains*	Enable the relevant security policies of all involved domains to be enforced and administered.

**Table 6:** The proposed requirements (left hand side) and the entities that they draw from Table 4 (right hand side).

Proposed Security Requirements Listed in Table 5	Mapping to the Relevant Entities in Table 4
*Light-weight solutions*	Energy awareness.
*Decentralized management*	Manageability, decentralization.
*End-to-end security*	Secure storage, secure communication, secure content, quality of service.
*Identity management*	Identity, anonymity.
*Privacy*	Privacy, location privacy.
*Mobility/Dynamic*	Mobility.
*Scalability/Incremental deployment*	Scalability, load balancing.
*Robustness/Self-healing/Reliability/Real-time/Data-freshness*	Resilience to attacks, real-time, data freshness, reliability, fault tolerance.
*Composition/Heterogeneity/Interoperability*	X
*Transiency/Ephemeral*	X
*Federation in administration domains*	X

**Table 7:** Some notable security requirements in different layers of an IoT security architecture discussed in Figure 1.

Architectural Layers	Notable Security Requirements (Discussed in Section 7)
Device sensing layer	Light-weight solutions/Support for composition, heterogeneity, interoperability/Robustness, self healing/Reliability, real time, data freshness, identity management/Mobility, dynamic.
Network management layer	Support for composition, heterogeneity, interoperability/Light-weight solutions/Scalability, incremental deployment/Federation of administration domains/ Mobility, dynamic.
Service composition layer	Robustness, self healing, reliability, real time, data freshness/Scalability, incremental deployment/ Composition, heterogeneity, interoperability/Decentralized management/Transiency, ephemeral.
Application layer	Robustness, self healing, reliability, real time, data freshness/Composition, heterogeneity, interoperability/Light-weight solutions/End-to-end security/Privacy.
User interface layer	End-to-end security/Mobility, dynamic/Scalability, incremental deployment/Composition, heterogeneity, interoperability/Decentralized management/privacy.
